# Unusual neurological presentation of second stage African trypanosomiasis in a young boy: a case report

**DOI:** 10.1186/s12887-022-03313-2

**Published:** 2022-05-12

**Authors:** Etedal Ahmed A. Ibrahim, Mohammed Gasm Elseed M. Elmahal, Khabab Abbasher Hussien Mohamed Ahmed, Elfatih A. Hasabo, Mohammed Eltahier Abdalla Omer

**Affiliations:** 1 Consultant Neurologist, National Center of Neurological Sciences, Khartoum, Sudan; 2 Associate Professor of Neurology, Department of Neurology, Faculty of Medicine, Al-Neelain University, Khartoum, Sudan; 3 National Center of Neurological Sciences, Khartoum, Sudan; 4 Faculty of Medicine, University of Khartoum, Khartoum, Sudan; 5 Teaching Assistant, Department of Internal Medicine, Faculty of Medicine and Health Sciences, Gadarif University, Gadarif, Sudan

**Keywords:** Human african trypanosomiasis, Trypanosoma, Second stage, Sudan

## Abstract

**Background:**

In South Sudan, sleeping sickness is a frequent condition caused by human African trypanosomiasis. There are two stages that are well-known. When the CNS is affected, especially with Trypanosoma gambiense infection, the early hemolymphatic stage and the late encephalitic stage have been observed, including mental, motor, and sensory symptoms. In this case, second-stage African trypanosomiasis manifested itself in an atypical neurological manner.

**Case presentation:**

A 16-year-old boy from South Sudan referred to Sudan National Centre for Neurological Sciences, Khartoum, Sudan suffering from non-convulsive status epilepticus, mental deterioration and behavioral changes for the last nine months. He was conscious but disorientated. Low hemoglobin concentration, elevated ESR, enlarged spleen and positive card agglutination test for trypanosomiasis was found in this patient. Electro-encephalogram (EEG) found an on-going generalized seizure activity. The patient showed improvement after management with carbamazepine and tonic.

**Conclusion:**

Our case highlights that late second stage African trypanosomiasis with neurological complications such as non-convulsive status epilepticus should be suspected in any patient who developed progressive cognitive decline and behavioral changes following long standing history of African Trypanosomiasis and routine Electro-encephalogram EEG is the best tool to diagnose non convulsive status epilepticus.

## Background

Protozoan parasites cause sleeping sickness, often known as human African trypanosomiasis (HAT). There are two types of the disease: an acute form produced by Trypanosoma brucei rhodesiense, which is mostly found in East Africa, and a chronic form caused by Trypanosoma brucei gambiense, which is mostly found in West and Central Africa [1].

HAT is divided into two stages: a hemolymphatic stage (stage 1or early stage) in which trypanosomes circulate in the blood or lymphatics, and a late stage in which the central nervous system (CNS) is involved [1]. *Trypanosoma brucei gambiense* is a slow-progressing parasite with an oligosymptomatic phase (few symptoms which are inconclusive, not specific and cannot be relied on when diagnosing a certain disease) that might span months or years [2].

Hemolymphatic stage – This is the first stage of the illness. Intermittent headaches, fevers, malaise, and arthralgia are common early signs of HAT infection. These signs and symptoms could be linked to waves of parasitemia and antibody generation. There may be hepatomegaly, especially splenomegaly, as well as widespread lymphadenopathy [3]. Pruritus, rash, weight loss, and face swelling are some of the non-specific symptoms that can occur [3]. Amenorrhea in women and impotence in men can both be caused by neuroendocrine disruptions [3] [4]. In the case of T. b. gambiense infection, this phase lasts around three years. T. b. rhodesiense, on the other hand, manifests as an acute illness with little differentiation between stages and can kill you in a matter of months [3]. This is a rare case of patient second stage African trypanosomiasis presented with unusual symptoms.

## Case presentation

A 16-year-old boy from South Sudan referred to Sudan National Centre for Neurological Sciences, Khartoum, Sudan suffering from non-convulsive status epilepticus, mental deterioration and behavioral changes for the last nine months. He was not malnourished and there was no fever, weight loss or lymphadenopathy. This disease affected the health status of the boy, and he became unable to communicate with his family and inattentive to his surroundings. This boy suffered from a decrease in school performance ended by leaving school. There were no motor or sensory symptoms, and no symptoms related to other systems were observed.

Past medical history is only significant to African trypanosomiasis three years ago which had been treated by combination therapy (full course of nifurtimox and eflornithine) for trypanosomiasis. His previous symptoms were fever, weight loss and excessive sleeping. Unfortunately, we lost his previous follow up after giving him the previous treatment. No family history of similar condition or neurological diseases were identified during taking clinical history.

### Physical exam

During physical examination, the patient was found conscious but disorientated to time, place and persons. Also, there was no lymphadenopathy or hepatomegaly but there was slight splenomegaly.

The boy scored zero in the abbreviated mental test score (a 10-point test, and score ≤ 8 indicate cognitive impairment). There were no signs of meningeal irritation. intact cranial nerves, normal fundal examination. Normal upper and lower limbs motor examinations. Sensations were intact. Vital signs were found stable and examinations for other systems were unremarkable.

### Work up

In the routine laboratory investigations for the boy, hemoglobin concentration was low (8.9 g\dl), mean corpuscular volume (MCV) was 65.5 fl and serum random blood glucose was 110 mg/dl. An elevated estimation sedimentation rate (ESR) was found during laboratory investigation (61 mm/hour). Other renal and liver function tests were normal. During blood film microscopy, no trypanosome were seen in buffy coat preparation, wet preparation, and thin blood film. Trypomastigotes were seen in the CSF and card agglutination test for trypanosomiasis was found positive in this patient.

### CSF Fluid analysis

We took CSF for analysis, and we found trypomastigotes and less than five white blood cells per milliliter. Level of glucose was: 8.7 mg/dl, and lactate dehydrogenase (LDH) was 27 u/l. CSF protein level was normal (29.3 mg/dl). No CSF centrifugation and microscopy was done for trypomastigotes in CSF.

### Radiological investigations

A normal magnetic resonance imaging (MRI) of Brain was found in this patient. Regarding ultrasound (US) for the abdomen, enlarged spleen with normal texture was observed.

**Fig. 1:**
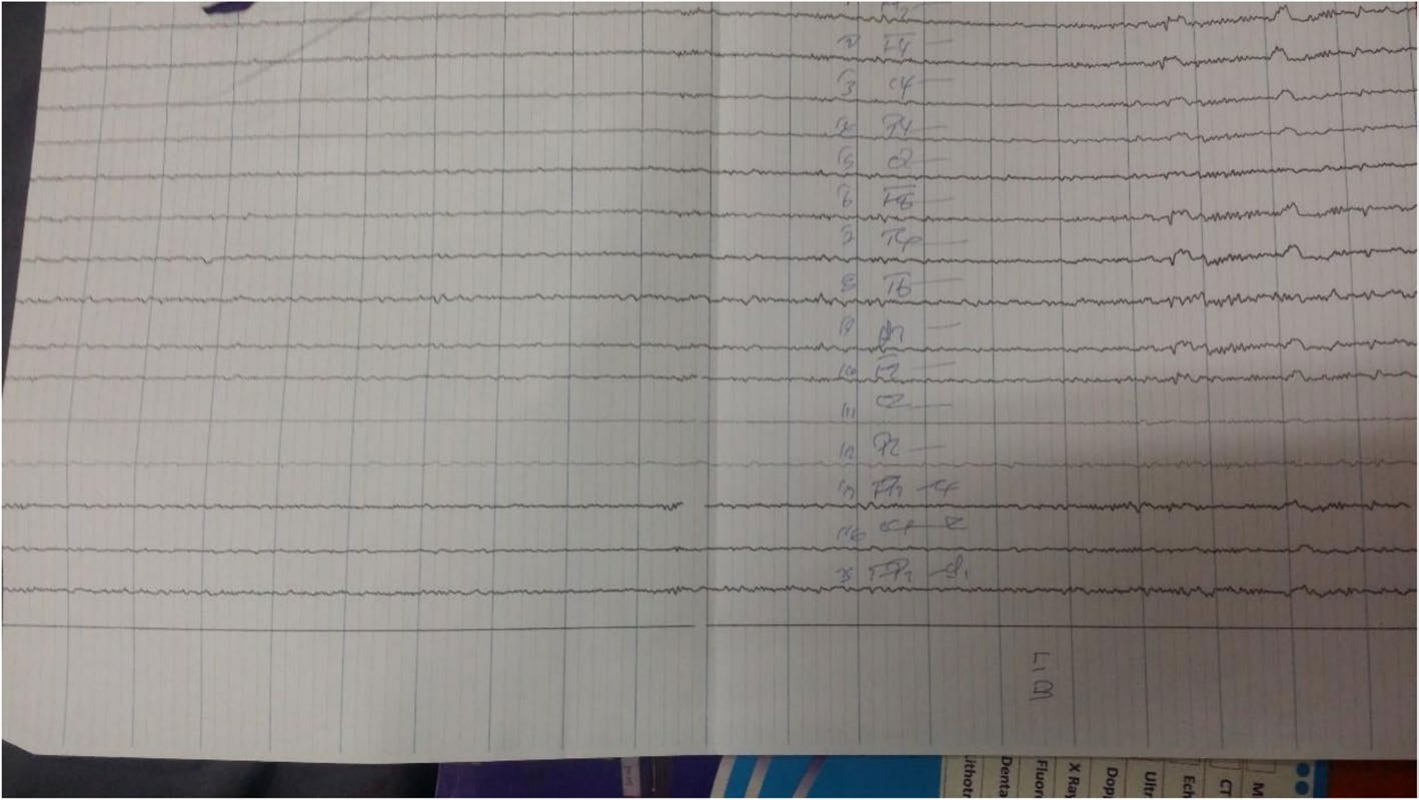
EEG finding of the patients shows generalized seizure activity

**Fig. 2:**
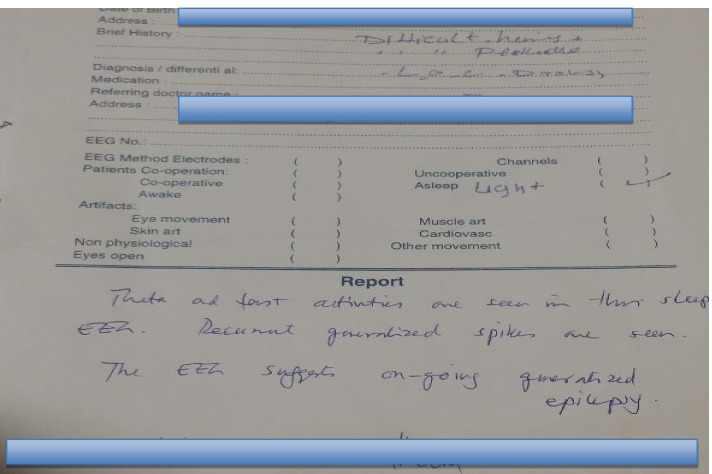
Printed EEG report of the patients by neurologist

Electro-encephalogram (EEG) suggested an on-going generalized seizure activity. (Fig. 1 and 2).

### Management

The boy was managed with a loading dose of intravenous phenytoin followed by a maintenance dose. Oral Carbamazepine 400 mg was given twice/day, tonics (ferrous sulphate 200 mg three times a day and folic acid 5 mg once daily) to improve his health status because we thought that his cognitive impairment had jeopardized his oral feeding, and combination therapy (Nifurtimox-Eflornithine) for African trypanosomiasis.

### Hospital course

This patient was admitted and managed for two months and he completed his combination therapy (Nifurtimox-Eflornithine) for African trypanosomiasis. After two weeks of treatment in hospital, he showed a remarkable improvement in his cognitive function and started to communicate with his family. After that, he was referred back to his country with a combination therapy (Nifurtimox-Eflornithine) for African trypanosomiasis if he encounters a relapse of the disease; because this treatment is not available in his home country (South Sudan) after separation from the republic of Sudan.

## Discussion and conclusions

In our case, we suspected late second stage African Trypanosomiasis with neurological complications such as non-convulsive state as the patient presented with progressive decline of his cognitive function and our suspicion was confirmed by EEG and supported more by remarkable cognitive improvement with anti-convulsant therapy. This case was confirmed by CSF examination.

These neurological symptoms occur in stage 2 (late presentation) of human African trypanosomiasis, and happens after spread of parasite via blood stream then enter blood–brain barrier to the nervous system causing these neurological symptoms [5]. Despite being managed with trypanosomiasis medications and having reassuring CSF studies showing less than five WBC cells and normal protein level, he developed late-stage disease and currently he has a positive card agglutination test trypanosomiasis and trypomastigotes in the CSF.

Unusual CSF findings are not typical and consistence with world health organization (WHO) guideline which stated a presence of more than 5 white blood cell and presence of trypanosome [6], and this could be explained by non-adherence to trypanosomiasis medications by the patient. Therefore, physicians should be aware that late-stage of African trypanosomiasis could present with unusual presentation such as deterioration in mental status and behavioral changes which lead to decline in performance.

The second stage of HAT need different drugs for management such as eflornithine and nifurtimox. In 2009, WHO recommended the use of combination drug that contains nifurtimox and eflornithine [7] based on the result of a large clinical trial [8].

Several adverse events were reported after administration of nifurtimox-eflornithine combination treatment (NECT). At least one adverse event (AE) was reported in (60.1%, 1043/1735) of total participants. Most common reported adverse events were gastrointestinal disorders (vomiting/nausea and abdominal pain), headache, and musculoskeletal pains [9]. Therefore a monitoring should be done for all patients who were receiving NECT for the management of second stage of HAT.

Also, Fexinidazole a 5-nitroimidazole derivative DNA synthesis inhibitor is considered a new drug for management of HAT [10].

Be believe that, this case of African trypanosomiasis is considered the first one describing late-stage of with these unusual neurological findings in Sudan.

Our case highlights that late second stage African trypanosomiasis with neurological complications such as non-convulsive status epilepticus should be suspected in any patient who developed progressive cognitive decline and behavioral changes following long standing history of African Trypanosomiasis and routine Electro-encephalogram EEG is the best tool to diagnose non convulsive status epilepticus.

Medical health professionals should suspect second stage African Trypanosomiasis with neurological complications such as non-convulsive status epilepticus in any patient who have history of African Trypanosomiasis and developed progressive cognitive decline.

Early referral of the patients with second stage African Trypanosomiasis with progressive cognitive decline to Neurology Centers with EEG facility might help to diagnose non convulsive status epilepticus in conjunction with early clinical suspicion of the condition will enhance the improvement of the prognosis. Further reporting of such cases is recommended.

## Data Availability

The datasets used and/or analyzed during the current study are available from the corresponding author on reasonable request.

## Abbreviations

CNS: Central nervous system
CSF: Cerebrospinal fluid
EEG: Electro-encephalogram
ESR: Estimation sedimentation rate
HAT: Human African trypanosomiasis
LDH: Lactate dehydrogenase
MCV: Mean corpuscular volume
MRI: Magnetic resonance imaging
US: Ultrasound
WHO: World health organization

## References

[1] Trypanosomiasis WHOEC on E and C of A, Organization WH. Epidemiology and control of African trypanosomiasis : report of a WHO expert committee [meeting held in Geneva from 16 to 23 October 1985]. Geneva PP - Geneva: World Health Organization; 1986. (World Health Organization technical report series ; no. 739). Available from: https://apps.who.int/iris/handle/10665/40346. 3099479

[2] Blum J, Schmid C, Burri C (2006). Clinical aspects of 2541 patients with second stage human African trypanosomiasis. Acta Tropica.

[3] Buguet A, Mpanzou G, Bentivoglio M. Human African Trypanosomiasis: A Highly Neglected Neurological Disease. In: Neglected Tropical Diseases and Conditions of the Nervous System. New York: Springer New York; 2014. p. 165–81. Available from: http://link.springer.com/. 10.1007/978-1-4614-8100-3_9.

[4] Büscher P, Cecchi G, Jamonneau V, Priotto G (2017). Human African trypanosomiasis. The Lancet.

[5] Kennedy PGE (2008). The continuing problem of human African trypanosomiasis (sleeping sickness). Annals of Neurology.

[6] Kennedy PG (2013). Clinical features, diagnosis, and treatment of human African trypanosomiasis (sleeping sickness). The Lancet Neurology.

[7] WHO Expert Committee on the Selection and Use of Essential Medicines (2009: Geneva S, Organization WH. WHO model list of essential medicines : 16th list, March 2009). Geneva PP - Geneva: World Health Organization; Available from: https://apps.who.int/iris/handle/10665/70642.

[8] Priotto  G , Kasparian  S , Mutombo  W , Ngouama  D , Ghorashian  S , Arnold U (2009). Nifurtimox-eflornithine combination therapy for second-stage African Trypanosoma brucei gambiense trypanosomiasis: a multicentre, randomised, phase III, non-inferiority trial. Lancet.

[9] Franco J, Pere S, Diarra, Ruiz-Postigo, Samo, Jannin. Monitoring the use of nifurtimox-eflornithine combination therapy (NECT) in the treatment of second stage gambiense human African trypanosomiasis. Research and Reports in Tropical Medicine. 2012;93. Available from: http://www.dovepress.com/monitoring-the-use-of-nifurtimox-eflornithine-combination-therapy-nect-peer-reviewed-article-RRTM. 10.2147/RRTM.S34399 PMC6067772 30100776

[10] Deeks ED (2019). Fexinidazole: First Global Approval. Drugs.

